# Intervention design for artificial intelligence-enabled macular service implementation: a primary qualitative study

**DOI:** 10.1186/s43058-024-00667-9

**Published:** 2024-11-26

**Authors:** Henry David Jeffry Hogg, Katie Brittain, James Talks, Pearse Andrew Keane, Technology Enhanced Macular Services Study Reference Group, Rashmi Kumar, Janet Lunn, Trevor Lunn, Rosemary Nicholls, Angela Quilley, Christine Sinnett, Gregory Maniatopoulos

**Affiliations:** 1 Research, Development and Innovation, University Hospitals Birmingham NHS Foundation Trust, Level 2 ITM, Queen Elizabeth HospitalMindelsohn Way, Birmingham, B15 2GW UK; 2 Department of Applied Health Research, School of Health Sciences, College of Medicine and Health, University of Birmingham, Birmingham, UK; 3 Moorfields Eye Hospital NHS Foundation Trust, London, UK; 4 Population Health Sciences Institute, Newcastle University, Newcastle Upon Tyne, UK; 5 Newcastle Eye Centre, Newcastle Upon Tyne Hospitals NHS Foundation Trust, Newcastle Upon Tyne, UK; 6 Institute of Ophthalmology, University College London, London, UK; 7 School of Business, Leicester University, Leicester, UK

**Keywords:** Artificial intelligence, Neovascular age-related macular degeneration, Implementation, Qualitative research, Theory, Theoretical approach, Machine learning, Clinical decision support, Ophthalmology, Retina

## Abstract

**Background:**

Neovascular age-related macular degeneration (nAMD) is one of the largest single-disease contributors to hospital outpatient appointments. Challenges in finding the clinical capacity to meet this demand can lead to sight-threatening delays in the macular services that provide treatment. Clinical artificial intelligence (AI) technologies pose one opportunity to rebalance demand and capacity in macular services. However, there is a lack of evidence to guide early-adopters seeking to use AI as a solution to demand-capacity imbalance. This study aims to provide guidance for these early adopters on how AI-enabled macular services may best be implemented by exploring what will influence the outcome of AI implementation and why.

**Methods:**

Thirty-six semi-structured interviews were conducted with participants. Data were analysed with the Nonadoption, Abandonment, Scale-up, Spread and Sustainability (NASSS) framework to identify factors likely to influence implementation outcomes. These factors and the primary data then underwent a secondary analysis using the Fit between Individuals, Technology and Task (FITT) framework to propose an actionable intervention.

**Results:**

nAMD treatment should be initiated at face-to-face appointments with clinicians who recommend year-long periods of AI-enabled scheduling of treatments. This aims to maintain or enhance the quality of patient communication, whilst reducing consultation frequency. Appropriately trained photographers should take on the additional roles of inputting retinal imaging into the AI device and overseeing its communication to clinical colleagues, while ophthalmologists assume clinical oversight and consultation roles. Interoperability to facilitate this intervention would best be served by imaging equipment that can send images to the cloud securely for analysis by AI tools. Picture Archiving and Communication Software (PACS) should have the capability to output directly into electronic medical records (EMR) familiar to clinical and administrative staff.

**Conclusion:**

There are many enablers to implementation and few of the remaining barriers relate directly to the AI technology itself. The proposed intervention requires local tailoring and prospective evaluation but can support early adopters in optimising the chances of success from initial efforts to implement AI-enabled macular services.

**Protocol registration:**

Hogg HDJ, Brittain K, Teare D, Talks J, Balaskas K, Keane P, Maniatopoulos G. Safety and efficacy of an artificial intelligence-enabled decision tool for treatment decisions in neovascular age-related macular degeneration and an exploration of clinical pathway integration and implementation: protocol for a multi-methods validation study. BMJ Open. 2023 Feb 1;13(2):e069443. https://doi.org/10.1136/bmjopen-2022-069443. PMID: 36725098; PMCID: PMC9896175.

**Supplementary Information:**

The online version contains supplementary material available at 10.1186/s43058-024-00667-9.

Contributions to the literature

- Artificial intelligence-enabled Software as a medical device (SaMD) is available for use in the management of neovascular age-related macular degeneration (nAMD), but no available research informs how it should be used.
- This UK-based study presents the first perspectives from patients, carers, clinicians, industry, regulators, policy makers, managers and commissioners on what can be expected to influence the implementation of AI into nAMD management and why.
- These insights inform an AI-enabled intervention ready for use or adaption by practitioners in their own macular service to help deliver benefits from available AI SaMD to their patients, their service and other stakeholders.

## Background

**Fig. 1:**
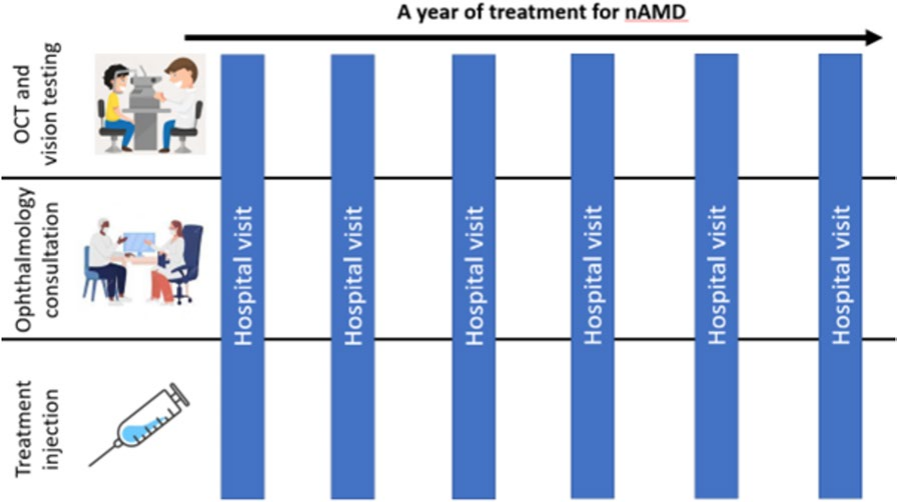
Schematic of typical neovascular age-related macular degeneration (nAMD) patient journey following diagnosis. OCT = Optical Coherence Tomography

Age-related macular degeneration (AMD) is the commonest cause of blindness in highly developed countries. The macula is the central part of the retina, responsible for central vision, and approximately 1% of Europeans over 60 years old are affected by a severe yet treatable form of AMD called neovascular AMD (nAMD) [1, 2]. nAMD treatment requires 4–12 injections into the eye each year, often for a decade or more (Fig. 1) [3]. Specialist clinicians base their judgements of the necessary frequency of injections on retinal imaging known as optical coherence tomography (OCT) [4]. Large numbers of nAMD patients, the need for regular sight-preserving treatment, and the finite ophthalmology workforce make avoidable sight loss through an imbalance of clinical demand and capacity a real risk [3, 5]. This makes nAMD services an important target for healthcare innovation [6].

There are several candidate solutions to this problem including new longer-acting drugs, drug delivery systems and telemedical workflows [7]. These intervention types are common in and outside of ophthalmology and attract significant investment from the established commercial entities who stand to profit from their adoption. Healthcare innovation through clinical artificial intelligence (AI) could complement any of these active innovation efforts. Whilst clinical AI continues to attract great interest and investment from industry, policy makers and academics, there remain relatively few examples of real-world AI-enabled workflows across healthcare and there are evidence-based concerns over the value of AI in those instances [8–12]. For eye care specifically, there are some global examples of successful AI-enabled diabetic retinopathy care pathways, but there is no precedent for AI-enabled real-world nAMD care [13]. This gap between the vision held for clinical AI by healthcare innovators and its reality on the ground may limit the long-term potential of clinical AI if a perceived failure to deliver persists.

Since 2022, at least five manufacturers have achieved regulatory certification for AI-enabled SaMD relevant to nAMD treatment [14]. Despite this apparent opportunity, there is currently no published precedent for the real-world use of these SaMD. This does not appear to be a limitation of the efficacy of the SaMD themselves or the Intended Use Statements against which they are certified. A recent qualitative systematic review of AI across healthcare settings suggests that improvements to the clinical evidence base should prioritise how specific clinical AI tools could best be implemented [15]. For AI-enabled productivity gains in macular services, this evidence could inform the design of an intervention to place any relevant SaMD into real-world care. Editorials and opinion pieces are the only contributions to the academic literature on the implementation of AI-enabled macular services, offering limited evidence and actionability [16, 17]. This study aims to explore what factors will influence implementation outcomes and why for AI-enabled nAMD treatment monitoring. Based on these factors, an intervention will be designed to give pragmatic guidance as to how the first attempts to implement AI-enabled macular services may be made.

## Methods

This explorative implementation study was described in an a-priori protocol and has been reported using TIDieR and COREQ reporting guidelines (Supplements 1 & 2) [18–20]. It is a primary qualitative study focused on a large teaching hospital in Newcastle upon Tyne, UK, delivering approximately 16,000 intravitreal injections for macular disease each year. Key features of the methods follow here, but greater detail and rationale is available in the supplement (Supplement 3) [21–28].

### Participant sampling

The sampling strategy was purposive, initially based upon stakeholder groups identified in a preparatory qualitative systematic review of clinical AI research [15]. Each participant was also asked to suggest other potential participants who might enhance study findings. Consensus in the research team that new participants had ceased to produce meaningfully novel insights for the first phase of analysis was awaited before recruitment was closed [29].

### Data collection

Semi-structured interviews were carried out, with participants choosing between an in-person (home or workplace) or videoconferencing approach (Supplement 4). If participants consented, interviews were recorded and pseudonymised prior to AI-enabled transcription (Otter.ai, Mountain View, CA, USA) and correction by the researcher. In the two instances that consent to record was withheld the interviewer took written notes.

### Data analysis

**Fig. 2:**
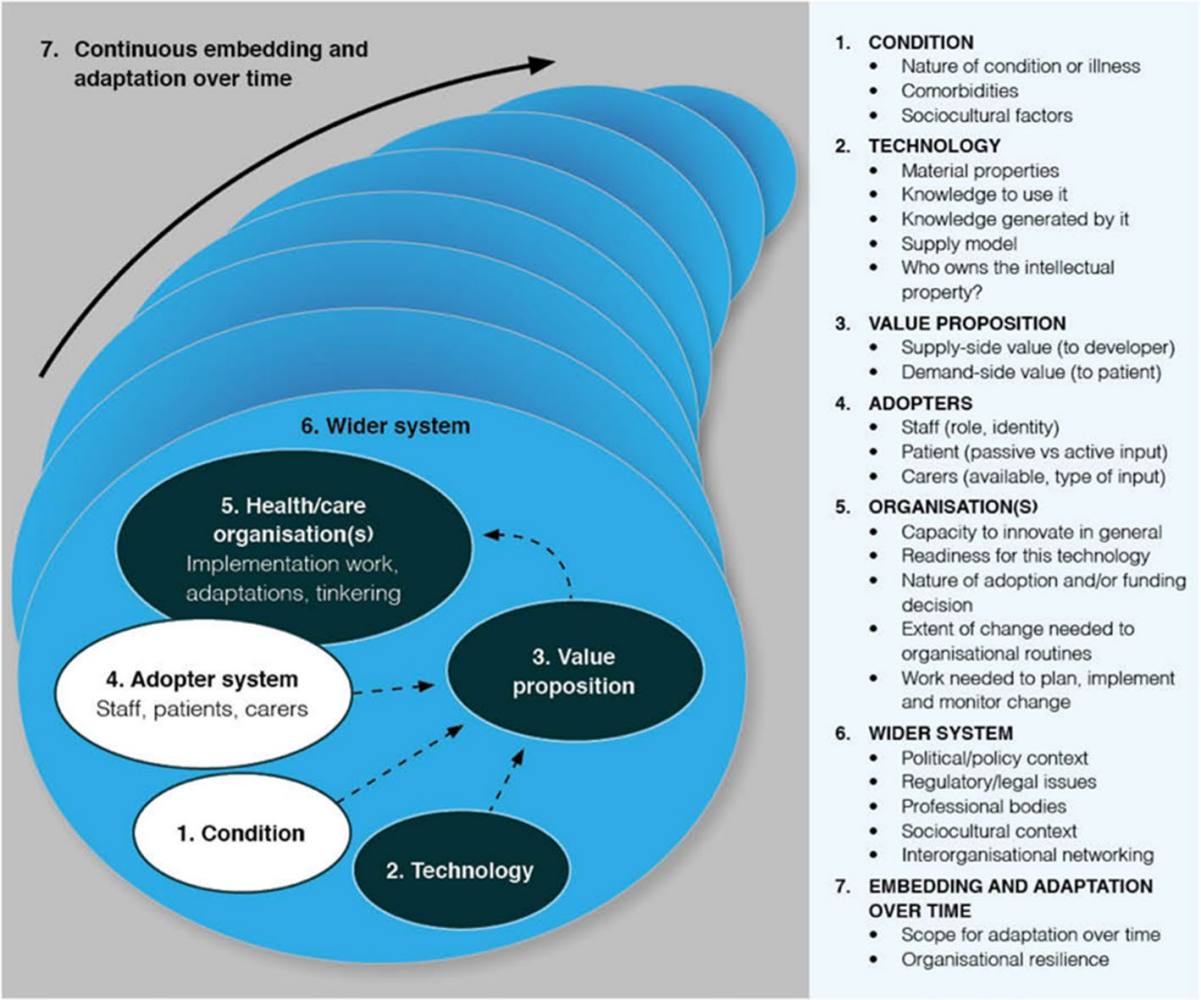
Schematic of the Non-adoption, Abandonment and challenged to Scale-up, Spread and Sustainability (NASSS) framework, reproduced from [21]

Data analysis was performed in two phases, both using NVivo (Release 1.2; QSR International). The initial analysis aimed to understand what factors could be expected to influence the implementation of AI-enabled macular services and why. The Non-adoption, Abandonment, Scale-up, Spread and Sustainability (NASSS) framework (Fig. 2) was used to guide this phase of analysis (Supplement 5) [21]. The analysis was concluded once recruitment had ended and the study reference group had no further suggested iterations on the analysis presented to them by the lead researcher.

**Fig. 3:**
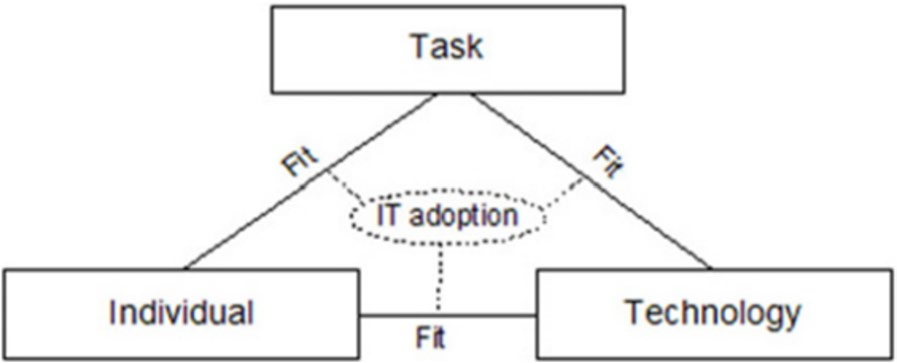
Schematic of the Fit between Individual, Technology and Task (FITT) framework, reproduced from [26]

The second analysis phase sought to propose an actionable AI-enabled intervention for the macular service studied. In practice, this intervention was planned to shape the study design of a simulated clinical workflow to be tested with practitioners following a separate diagnostic accuracy study. However, this intervention and the logic underlying its design were also intended to be adaptable for other macular services seeking guidance on how to enact AI implementation. Both original data and findings of the initial analysis were used in this second analysis The Fit between Individuals, Technology and Task (FITT) Framework was identified as a relatively simple, action-oriented model, focused upon the modifiable elements of an intervention (Fig. 3) [26]. These qualities were well suited to the practical goal of this secondary analysis to provide accessible and actionable guidance to a range of different practitioners on how they should implement AI. This second analysis concluded once recruitment had ended and the study reference group had no further suggested iterations on the intervention and rationale presented to them by the lead researcher. This intervention was subsequently validated through parallel roundtable discussions at a public engagement event, with no further call for change at this pre-implementation stage (Supplement 6).

## Results

Thirty-six individuals consented to take part in 35 semi-structured interviews (Table 1). Six of the participants were patients (Table 2). Twenty-six interviews were conducted face-to-face, with nine conducted by videoconferencing software. The median length of interviews was 47 min (range 00:27 – 01:24). Fourteen patient and five other individuals invited to participate were unable to find time or declined to do so.

**Table 1:** Characteristics of non-patient participants by stakeholder group

Stakeholder group	Number of participants	Characteristics
Carers	2	1 son^a^ and 1 daughter of 2 separate patients; 1 cohabiting, 1 living nearby
Charity professionals	2	1 regional manager, 1 national director
Clinicians	13	1 retinal consultant, 2 registrar ophthalmologists, 2 advanced nurse practitioners, 2 hospital/community optometrists, 2 community optometrists, 1 GP partner, 2 ophthalmic photographers, 1 social work-hospital liaison
Commissioners	2	1 ICB clinical commissioning lead for ophthalmology, 1 ICB commissioning project manager
Industry professionals	4	2 imaging manufacturer representatives with national and international roles, 2 software manufacturer representatives with national and international roles
Managers	5	1 directorate manager, 1 outpatient clinical manager, 1 independent sector provider manager, 1 macular service administrator, 1 service innovation manager
Policy makers	1	National level policy maker
Regulatory professional	1	Senior regulatory consultant

*ICB* Integrated Care Board

^a^Did not consent to interview recording

**Table 2:** Characteristics of patient participants

ID	Gender	Ethnicity	Age	Miles from hospital
1	Male	White British	70s	31
2	Female	White British	60s	5
3	Male	White British	80s	5
4^a^	Female	White British	90s	13
5	Male	White British	90s	21
6	Female	White British	80s	3

*ICB* Integrated Care Board

^a^Did not consent to interview recording

### Factors influencing implementation outcomes

#### Domain 1: condition

As a condition, hospital eye clinicians found nAMD relatively simple to treat and expressed frustrations over the amount of input that serial treatment and monitoring episodes required from them. Despite its simplicity, its management was seen as a specialist concern and primary care participants were clear that ophthalmologists’ held sole responsibility for it.

*“Our relation to eyes is almost similar to our relation to teeth, we’re better than no one, but we’re not the guy.” [General Practitioner]*

Consultant ophthalmologists’ oversight of elements of nAMD care in the community was proposed by primary care participants. Many participants from different stakeholder groups alluded to the current secondary care ownership of nAMD management as a barrier to freeing up ophthalmology capacity and making better use of a larger community workforce. Another problem raised with the centralisation of nAMD care was the high prevalence of frailty and co-morbidity among people with nAMD.

*“They’re old these people, and they’ve got sight difficulties. So, it’s going to be quite daunting, getting on a bus and getting from the bus station up to the RVI [hospital].”* [Community optometrist]

Patients and hospital clinicians also highlighted the challenges that a clinic visit for nAMD pose for people with such comorbidities as patients must navigate various rooms, chairs and couches through a single visit.

#### Domain 2: technology

All potential users of the AI technology held relatively abstract perceptions of its nature and role in their work. At a high-level, expectation of reduced patient-clinician dialogue, high performance in image interpretation and enhanced clinical productivity was commonly voiced across stakeholder groups.

*“It’s nice to see a person, you know? If you had this machine and then it would print out “She doesn’t need any more [injections],” or, “She does.” It’s not like asking the doctor*” [Patient]

When talking in greater detail about the AI technology, participants would commonly conflate different uses or types of AI, e.g. AI used to detect nAMD activity or other ocular and systemic diseases. The regulatory participant pointed out that such flexibility in an AI medical device would not be permissible within its certification. This highlighted risks of use case-drift from clinicians and misinformed fears and expectations from patients. There was a range of expectation over how much autonomy would be assigned to AI. If meaningful evidence of its trustworthiness was shared with them, most participants seemed accepting of eventually allowing independent AI decisions to be made for a proportion of treatment monitoring episodes.

*“…say that this AI technology is good enough to reliably, and with a very good true positive and very low false positive result, say that, “This patient needs to be seen. This patient could be seen in eight weeks. This patient can have injections deferred now.” I think that would be favourably looked upon [by commissioners].”* [Commissioner]

The feasibility of some attribution of responsibility to AI appeared important as the business case for AI-enabled macula services was seen as stronger in more autonomous AI-enabled scenarios. Industry participants also highlighted the importance of interoperability with an adopting organisations digital infrastructure to make adoption financially, technically and operationally feasible.

#### Domain 3: value proposition

The value proposition described by participants went beyond productivity and effectiveness gains to encompass financial, clinical and inter-organisational benefits. One commissioning participant explained that financial incentives in the current commissioning system lay with providers using AI to reduce their costs per unit of billable service, rather than expecting higher tariffs for AI-enabled care.

*“On Saturdays you could have four consultants instead of one consultant and then juniors. So, because we just need to get the patients through, it potentially could be really expensive. But the hospital is happy to keep on going, because obviously we can’t put the patients at risk.”* [Manager]

This clinical manager felt the financial value proposition of improving productivity would help address their current problem around expenditure on staff overtime to control clinic waiting lists. Besides costs, participants broadly saw AI as an opportunity to improve the transparency of clinical management. This came from patient, carers and clinician participants who shared examples of dissatisfaction from limited clarity on current disease states or the rationale for decisions.

*“So being aware of what's going on and why and being able to discuss it is important. So what should happen when I go to the NHS is that they should do what the optician does, say 'Well, here it is [patient’s name]. This is what has occurred and how it has changed’.” [Patient]*

Clinicians performing injection-only clinics also reported frustrations where ambiguous documentation from clinical colleagues disrupted their workflow, requiring them to go and seek clarification in person. They assumed that AI-enabled workflows would mitigate this, expecting that the clinical rationale underlying decisions would be more standardised. This standardisation was expected to hold additional value for care pathways which span primary and secondary care providers.

#### Domain 4: adopters

Patients seemed assured that the service’s ‘human touch’ would persist regardless of AI due to the need for clinicians to administer injections. Many participants anticipated that AI could improve limited clinic capacity, which was a problem clearly perceived by some patients.

*“So, I think that is probably the most stressful thing. It [phoning to check appointment booking] is just something that I need to do. I have found almost every time the last 10 or 11 [clinic appointments].”* [Patient]

There was clear hope from patients that an AI-enabled service could lessen this burden, not only through decentralisation, but by facilitating shorter more predictable appointment lengths and increased appointment availability. Ophthalmologists appeared to welcome task shifting toward AI as they viewed OCT interpretation in nAMD as a low complexity task requiring little personal exposure to for achieving and maintaining clinical competence. Nursing and optometry staff also appeared broadly positive about AI implementation for this use, suggesting it may lessen their current dependence on medical colleagues to resolve ambiguous or complex cases. They expressed some concerns that highly automated AI uses might displace them from their nAMD decision making role.

*“But, I suppose with AI, that the good thing is at least there is some research backing the fact that we have worked out what the specificity and the sensitivity of all these things are for certain conditions. However, we have trained them [junior clinicians] up by looking at a set of images and going through them together, and then we have given them feedback. But we have not actually measured how well they perform.”* – [Consultant retina specialist]

This consultant’s reflection on their personal risk from assuming responsibility for the unquantified quality of junior colleagues’ decisions lends some legitimacy to the professional threat that allied health professionals alluded to.

#### Domain 5: organisation

Although operational staff in adopting organisations appear to be the decision makers on AI implementation, they and other stakeholders are strongly influenced by consultants. This was despite ophthalmologist participants reporting a low familiarity with the technology and limited competency in evaluating it. Industry and management participants highlighted that the strong influence of consultants’ views can be problematic in organisations as individual consultants’ perspectives can disproportionately impact implementation efforts.

*“People distrust things and, let’s face it, consultants can be a bit, “Nobody can do this as well as me.” I love them dearly but…”*- [Manager]

Several professional stakeholders also pointed to the availability of adequately trained Information Technology (IT) professionals as a key enabler for an organisation to manage the risks and technical challenges associated with providing AI-enabled care.

*“…you end up with a couple of groups of people in NHS IT. You end up with people who are very passionate… [and] you find people who might apply on the regular for job openings at, let's say, Cerner or Deloitte, but not quite making the cut. That workforce mix doesn't always lend itself to being the most efficient at implementing the types of things [clinical AI] we've been talking about”* [Industry professional]

Concerns over the suitability of the NHS workforce was echoed by another industry participant who presumed that technical and operational expertise from third party consultants would be required if NHS organisations were to successfully implement AI-enabled care. Despite these apparent limitations in organisational readiness, NHS managers and commissioners all pointed to improving the productivity of nAMD services as a strategic priority that AI could help to address.

*“I think if any Trust anywhere in the country says that they are managing it [nAMD care] successfully they are not telling the truth, because almost all of them are firefighting…”* [Commissioner]

#### Domain 6: wider system

It was common for all stakeholder groups to volunteer negative associations with other forms of AI (and often robotics) from public media and science fiction sources. Even following further explanation of the technology on which this study focused, these associations often resurfaced within discussions and appeared likely to influence peoples’ perceptions of AI-enabled care.

*“I think rightly so we should be frightened of it [AI]. You know, we've all watched films like ‘9’... and I think we do need to be very respectful of that. And of people's fears of it as well. You know, look at the response to [Covid-19] vaccination and the amount of distrust that it uncovered.” [Manager]*

Patient and charity participants highlighted that information and engagement during the design and early implementation of clinical AI interventions could help to build trust in new care pathways. Community optometrist participants also raised concerns that different groups of patients may find it more challenging to access nAMD services. These groups were mainly people for whom repeated journeys to hospital were particularly challenging (more comorbidities, social isolation, rurality or financial pressures). For that reason, greater decentralisation of nAMD care, whether facilitated by AI implementation or not, was seen as a likely means to improve service equity. Charity professionals self-identified their role as surfacing the patient voice on issues such as service equity and advocating for them across the various stakeholder groups driving innovation.

*“We do work quite closely with NICE and we are engaging more with different parts of the NHS and some of the strategy development around these pathways being designed.” [Charity professional]*

#### Domain 7: embedding and adaption over time

Most participants highlighted a sustained need to improve NHS capacity for nAMD care. Participants expected this need to increase over time. Similarly with AI as an innovation, industry and NHS professional participants all felt there to be a high demand for AI-enabled healthcare innovation.

*“I would say that ministerially and in other aspects, there is a need to get something [an AI-enabled care pathway] out because there are short-term objectives of political will.”* [Policy maker]

This national policy lead explained the political drive to achieve some widely recognised examples of successful clinical AI implementation. They also emphasised that these examples were likely to be achieved in clinical areas with pre-existing high digital maturity or need for change. Alongside other perspectives from industry and commissioners, these questions over the scalability of AI benefit across the NHS in general suggested that the sustainment of the current demand for clinical AI should not be assumed. This risk was reflected by industry participants, all of whom were employed by large companies invested in clinical AI strategies. These strategies seemed to be oriented more toward system solutions (e.g. cloud platforms) capable of aggregating the success of a range of AI products.

*“You need to work with the existing system that you have, which is massive, convoluted, complicated, and is regulated both by government and other bodies. And introducing changes to such an organism is not easy at all. So, we will see it [AI implementation] as an iterative process.”* [Industry professional]

This international strategic lead for a MedTech company shared their appreciation for the complexity of implementing AI and suggested that better informed strategies would become possible as outcomes from the current wave of interest in AI are evaluated.

### Intervention design

#### FITT framework – task

**Fig. 4:**
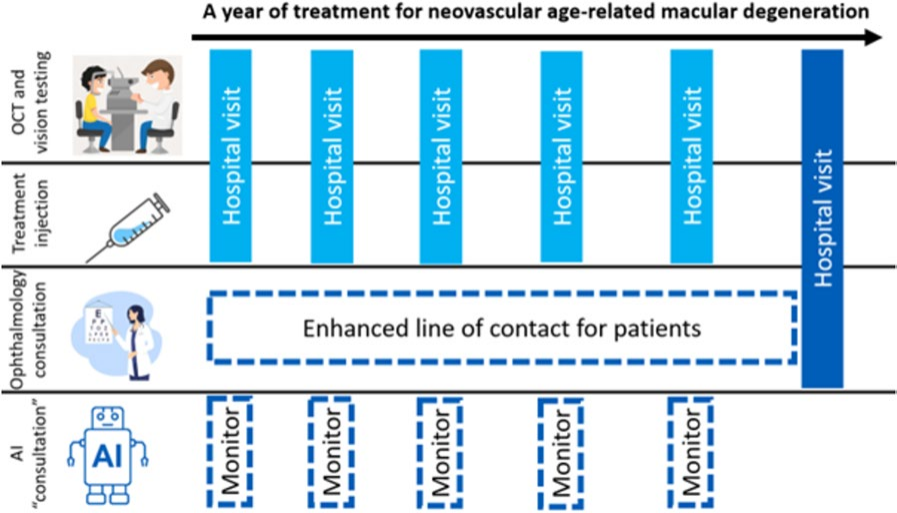
Schematic of proposed AI-enabled intervention for nAMD treatment monitoring

The data analysis indicates that the AI technology should focus on treatment monitoring for patients with an established diagnosis of nAMD as this accounts for the majority of the problems in the service, high volume low complexity work. This AI should incrementally assume relatively autonomous decision-making roles (rather than purely decision-support) for at least some clinical decisions (Fig. 4).

Points in the care pathway that deal with clinical and interpersonal complexity (such as diagnosis or screening for ocular comorbidities) were seen to require specialist clinician input. One patient emphasised that he “*wouldn’t like to go straight from AI diagnosis to treatment*” but did value the prospect of extra clinician time being focused on diagnostic consultations. “*I mean, no one spoke about this. They just said, oh, you’ve had a [macular] haemorrhage*”. AI should instead target the high volume and low complexity situations in nAMD care, which one ophthalmology trainee described as “*when we are comfortable with the diagnosis and they are on a treatment*” and “*we just need something that can crunch [data]*”. Due to existing treatment protocols, the risk of patients losing vision from automating a proportion of such decisions was seen as low. With adequate explanation and safety-netting from clinicians, patients appeared very accepting of dropping consultations from most of their treatment appointments, “*that wouldn't bother me if they thought I didn't need to see them all the time”* [Patient].

Setting the AI this relatively independent task would help to clarify the productivity value proposition of AI-enabled treatment monitoring. Making such a clear value proposition would strongly incentivise payors, with one commissioner explaining that “*any innovation that would reduce your backlog by 50%, by 25%, they [the board] would just commission it*”. Whilst this relatively high level of automation appeared to be a desirable end-goal for an AI-enabled intervention, it will be important to approach it iteratively in partnership with patients, clinicians and managers. Some patients were willing to trust AI if their clinical team appears supportive, but others want direct access to accessible of evidence of its performance. One patient with a scientific background said they would want to see that AI decisions on their own prior imaging agreed with their clinician’s. Only then would they permit autonomous AI decisions “*for the next two or three [treatments] and then I will come down and*” have another consultation. Clinicians echoed this need for personal evaluation of technology and a gradual trust-building process. A manager reflected on the positive influence of a local evaluation on subsequent treatment innovation, anticipating that both for patients and staff, such a transparent evaluation of AI-enabled nAMD monitoring would, “*give you that sense of pride and ownership*”.

#### FITT framework – Individuals

The data analysis suggests that consultant ophthalmologists should remain accountable and contactable to satisfy the need for specialist oversight of nAMD care. They can achieve this whilst reducing their contribution to treatment delivery and patient consultations (Fig. 4). A larger pool of allied health professionals should be recruited to lighten individuals’ injection duties. Medical photographers should be responsible for operational aspects of AI use.

An AI-enabled intervention should enable managers’ broader goals “*to diversify our workforce, so we have lower qualified staff*” [NHS hospital manager] to take monitoring tasks away from scarce and expensive ophthalmologists. A General Practitioner warned this should not compromise patients’ sense of connection with consultants as their patients already report low satisfaction from seeing specialty trainee doctors, *“if it’s, “I was only seen by the algorithm,” I can imagine that being an even lower drop*”. Ideally this would be addressed by prioritising consultants’ time for initial diagnostic appointments as patients enter the service. Consultant time would also facilitate the supervision or generation of replies to concerns or queries submitted by patients through additional lines of communication. The intervention should also free up allied health professionals and trainee ophthalmologists to spend more time communicating with patients and using their wider competencies outside of macular care. “*I don’t see it as a good use of my time after my 10 years of training to look at an OCT for someone who’s already got a diagnosis…. I mean, why*?” [ophthalmology trainee].

Interoperability issues within and between the various health technologies involved require a staff group to take responsibility for applying the AI and documenting its outputs. Within the hospital setting, hospital photographers were accepting of this role and endorsed by other stakeholders. One ophthalmic photographer shared that the favourite part of their current role was “*when you help diagnose something*” whilst a separate ophthalmic photography lead felt his staff would be very happy to take on responsibility for AI as “*it fixes you very firmly in a critical role”*. When asked who they thought should take responsibility for the AI one patient answered, “*I think it should be the people that do the photographs now*”. Whilst most patients wanted overall responsibility for the AI to be held by a consultant ophthalmologist, none required that relaying or explaining AI outputs involved ophthalmologists and many preferred it not to.

Having a member of the team dedicated to absorbing the technical complexity of AI integration also maximises the productivity of clinicians focused on delivering injections. This is because for injection staff, “*I'm not in the role of a [pay band] 7, where I'd be reviewing patients. I'm interested in what the diagnosis is, bang, bang, bang. What drug? What eye? When to bring them back?”* [Advanced nurse practitioner]. The output of the AI that photographers might add to the electronic record should reflect these practical requirements from injectors and other stakeholders.

Whilst injection duties were welcomed by nurse and optometrist participants in moderation, it was felt important to keep a good “*balance of the injections so you’re not all stuck on injections all day*” [Hospital optometrist]. Along with considerations for clinician satisfaction, staff with consultation and examination competencies are higher paid than those with just injection competencies. Applying large amounts of their time to perform duties within the scope of lower paid staff would not be cost effective.

#### FITT framework – technology

The data suggest that the commercial risk of trying to lead competitors in a changing market mean that initial SaMD products are likely to come from smaller commercial vendors. To address the problem of complex existent digital infrastructure and the clinician time absorbed in navigating it, the SaMD will need to be compatible with adopters’ Picture Archiving and Communication System (PACS), electronic medical record (EMR) and imaging file formats. SaMD outputs will ideally be smoothly integrated into clinical, administrative and other professionals’ workflows.

Although AI for nAMD decision support from OCT analysis could be supplied to providers as a stand-alone software to be integrated onsite, *“there will be a lot of work on the hospital side to do that integration”* [Medtech industry professional]. Such an approach with AI was also described as risky as subtle changes in input data “*could impact the result in ways that are unintended, so the software needs constant monitoring”* [Medtech industry professional]. Industry participants were sceptical of most NHS organisations’ capacity or capability to undertake that kind of integration and monitoring work. Consequently, the SaMD should access provider data on the cloud, as *“having a cloud-based solution lets the vendor be involved in the ongoing surveillance of the performance of their product”* [Medtech industry professional].

The larger MedTech companies represented in the study appeared to be avoiding developing the SaMD themselves, because such a company “*doesn't want to be burdened with all the regulations and all the issues of developing and clearing such products”* [Medtech industry professional]. They appeared to focus on systems solutions, offering products that are likely to address the interoperability issues between imaging formats, PACS, cloud platforms and SaMDs. This is particularly positive for scalability as like its competitors, the ophthalmic imaging company represented “*have a really great installed base of diagnostic imaging systems and PACS and EMR in the case of the UK”.*

The SaMD remains a foundational part of the intervention however, and the manufacturer “*needs to be a legally registered entity and take legal responsibility for the ownership, distribution, sale and eventual killing of the device*” [Regulatory professional]. The regulatory participant in the study was not aware of any NHS organisation acting as manufacturer for SaMD to date. Industry participants pointed to a handful of small companies who supply SaMD to their cloud platform. The nature of this arrangement adds some complexity “*where the [hospital] site that uses it would have to sign a contract”* [Medtech industry professional] in addition to its procurement of the cloud platform from the larger MedTech company.

Ideally, the digital infrastructure which the SaMD interacts with should allow smooth integration of its outputs with the workflow of different professional groups. Clinicians want some sense of “*how happy it is with the answer it has given you*” and “*would definitely prefer to know exactly why the machine comes up with*” [ophthalmology trainee] a given recommendation. Integration with appointment scheduling facets of the EMR, which were not interoperable with clinical components of the EMR, should also be pursued. One NHS administrative manager thought that an AI-enabled workflow could improve the safety and efficiency of the service’s work as they thought “*it could do away with a lot of having to monitor the appointments”* and anticipated more accurate forecasts and management of clinical capacity.

## Discussion

### Principal findings

This study has provided evidence on what can be expected to influence the implementation of AI-enabled macular services and why. It has also applied theory to make actionable recommendations from this evidence to support practitioners as they make the first implementation attempts. Even within this single-centre study, many interdependent factors are shown to influence implementation outcomes and the nature and extent of those influences will vary over time and between centres. Examples of this interdependency include the value proposition of moving care from the hospital to community settings influencing the individuals likely to adopt the intervention, the high volume of clinical activity related to treatment monitoring for the condition influencing the task which the technology is developed to perform, and the adaption over time of NHS digital infrastructure influencing the optimal delivery model of the technology. The pragmatist perspective underlying this study does not seek to deny this complexity, or claim universal value for the exact intervention proposed. The generalisable value of this study for other macular services can instead be realised through its transparent reporting. The data and rationale underlying the proposed intervention enable local practitioners to mobilise insights of their own macular service and shape their own place-based intervention. These insights may concern local workflows, patient populations, digital infrastructure or other considerations.

### Comparison with wider literature

An intentional difference of this study when compared against much qualitative clinical AI research is its transparency over the process underlying theoretical approach selection, and its rationale [25]. It is intended that by sharing both process and rationale, other researchers can be more informed in making theory selections for their own work and through collective effort the field may be more effectively progressed. For example, a comparable qualitative study of a rule-based AI tool for nurses in China sought to characterise barriers and facilitators to its implementation. The authors used FITT to guide data collection and analysis but found its lack of consideration for organisation and system level factors limiting [30]. The relevance of such factors to implementation are well established and may have been more effectively illuminated through different theoretical approaches. The NASSS framework was considered as an applicable mapping tool for the present study for this reason [21]. In the context of the present study’s aim however, NASSS alone was not felt to offer the desired actionability of findings. This prompted a review of theoretical approaches used in clinical AI research and the selection of FITT to provide actionable direction for a range of different implementation practitioners [25].

A key finding from this study is the need for accessible and credible evidence that implementing AI-enabled macular services will not risk the sight of nAMD patients. This echoes qualitative research of nAMD patient priorities elsewhere [31]. Research to produce this interventional evidence in nAMD treatment monitoring is in progress [20, 32]. If this or other work produces evidence of clinical and cost effectiveness, the present data suggest that stakeholders will be broadly welcoming of AI-enabled macular services.

The ability to adapt the intervention proposed in this study may be key, as existent regulatory approvals do not permit autonomous applications of AI within macular services [14]. This conflicts with many study participants’ presumption that productivity gains from AI would depend on its ability to automate at least some decisions. If an intervention can be crafted which employs the SaMD for decision support roles which reduce cautious overtreatment, then productivity gains from AI-enabled macular services may still be feasible. The overlay of current clinical decision heuristics from nAMD management on reviewable AI-generated OCT segmentations also enables ‘AI interrogation practices’ [33]. This ability to interrogate AI has been shown to promote acceptance in other medical image analysis tasks [34].

### Limitations

A potential limitation of this study is its narrow scope of investigation centred on a single site for a disease-specific AI application. The transparency of analysis and reporting is intended to mitigate against this, to allow readers to interpret findings to support AI-enabled innovation of other macular services. Another limitation was the lack of direct experience from study participants with AI-enabled macular services, which was unavoidable at the time of the study [16]. This may limit the relevance of insights that participants can offer for real-world care. Further work should evaluate how the proposed mechanisms for implementation outcomes translate in the varied contexts of real-world AI-enabled macular services. Such work will help to more robustly answer the wider question for AI-enabled macular services of “what works, how, for whom, in what circumstances and to what extent?” [35].

## Conclusions

Many factors will influence the implementation AI-enabled macular services. The potential value proposition for AI in this context is widely accepted by key stakeholders and extends far beyond the motivating opportunity to improve capacity in nAMD services. This study proposes an actionable intervention with a transparent evidence-base. This takes the form of an alteration from the current nAMD treatment pathway to reduce the number and increase the quality of face-to-face consultations whilst using AI decision support to improve the productivity of clinicians making treatment decisions. This can be expected to improve patient and clinician experience whilst also increasing service efficiency. This intervention acts as a starting point for prospective evaluation through clinical trials and place-based service evaluations.

## Supplementary Information

Supplementary Material 1. S1. TIDieR checklist.

Supplementary Material 2. S2. COREQ checklist.

Supplementary Material 3. S3. Detailed methods.

Supplementary Material 4. S4. Example topic guide.

Supplementary Material 5. S5. Example NASSS narrative summary.

Supplementary Material 6. S6. Engagement event outline.

## Data Availability

Codebook from pseudonymised interview transcripts can be made available from the corresponding author on reasonable request through governance processes approved in the ethical approval.

## Abbreviations

AI: Artificial Intelligence
AMD: Age-related macular degeneration
nAMD: Neovascular age-related macular degeneration
COREQ: Consolidated criteria for reporting qualitative research
EMR: Electronic Medical Record
FITT: Fit between individuals, technology and task
ICB: Integrated care board
IT: Information Technology
NASSS: Non-adoption, abandonment, sustainability, scale-up and spread
NHS: National Health Service
OCT: Optical coherence tomography
SaMD: Software as a medical device
TIDieR: Template for intervention description and replication
UK: United Kingdom
USA: United States of America
