# Supplementary material for: Intervention design for artificial intelligence-enabled macular service implementation: a primary qualitative study
Source: Implement Sci Commun. 2024 Nov 26;5:131. doi: 10.1186/s43058-024-00667-9 (PMC11600873; doi:10.1186/s43058-024-00667-9)
Supplement: Supplementary file 3 — Supplementary Material 3. S3. Detailed methods. [file 43058_2024_667_MOESM3_ESM.docx]

# Detailed methods

## Participant sampling

A qualitative systematic review of primary AI implementation research completed in preparation for this work abstracted five key stakeholder groups from the literature; clinicians, public/patients/carers, healthcare managers/leaders, regulators/policy makers and industry professionals.(15) The purposive sampling in this study aimed to represent each of these five stakeholder groups, understanding their high level of abstraction and pursuing opportunities to expand the granularity of each stakeholder group where it appeared valuable and feasible. Beside the lead researcher’s own clinical and academic experience, the purposive sampling was also informed by study participants, who were each asked to suggest other important informants at the close of their interview. Sampling was also informed by the study’s advisory and reference groups, which held a range of public, patient, carer, clinician and academic perspectives. These groups imparted a drive to maximise the diversity of participants. No compensation was offered to either public or professional participants in the research.

## Data collection

Semi-structured interviews were selected over a survey tool as the research team felt the evidence gap around AI macular services meant that too little was known about what questions would be important to adopt the more prescriptive approach to questioning offered by surveys. This need was felt to outweigh the potential for more generalisable findings from a larger scale of recruitment. Semi-structured interviews were also preferred over focus groups to allow more in depth and convenient explorations for participants. It was also anticipated that eligible individuals may either be relatively frail and be disincentivised to engage in research away from home, or in very busy senior roles where social and political forces may influence the perspectives they wished to share in a group setting. These considerations also pointed to a 1:1 interview approach. This preference for interviews over focus groups reduced the likely scale of study recruitment and the nature of the data, which was free from direct peer influences.

All interviews were conducted by the lead researcher (HDJH), an ophthalmologist with postgraduate training and experience in qualitative research. Method design and data analysis and interpretation were supported by the study reference group, including two senior qualitative researchers (KB and GM). The lead researcher had pre-existing professional relationships with some participants but had not been involved in the care delivery of any patient participants. For these semi-structured interviews participants were asked if they consented for the interviews to be recorded. If so, recordings were sent to AI-enabled transcription (Otter.ai, Mountain View, CA, USA) service where the researcher directly reviewed and corrected each transcript against the recording. These transcripts were treated confidentially and pseudonymised prior to analysis. Where participants refused for the interview to be recorded (one patient and one carer only), the interviewer took brief notes through the interview itself and then made a voice recording in private immediately after the interview to capture insights as fully as possible.

The questions posed in these semi-structured interviews drew on the contributions of the participants, but also on pre-prepared topic guides (Supplement 4). The initial topic guide was informed by the findings of the aforementioned qualitative systematic review and the adapted Non-adoption, Abandonment, Scale-up, Spread and Sustainability (NASSS) framework which supported the best-fit framework synthesis method used.(15,21) Before starting interviews the topic guide was reviewed multiple times by different members of the study group (patient, public, carer and senior qualitative researcher representatives) to ensure clinical or technical jargon was avoided. Subsequently, the topic guide was re-iterated prior to each interview to take account of insights from emerging data and the stakeholder group which the next participant came from.

## Data analysis

The first phase of analysis aimed to understand what factors could be expected to influence the implementation of AI-enabled macular services and why. Members of the research team with qualitative expertise felt this aim aligned with the offering of a determinant framework designed to accommodate factors across individual, organisational and system levels.(22) A number of commonly used theoretical frameworks could have satisfied this including the Consolidated Framework for Implementation Research (CFIR) or the Technology, People, Organizations and Macroenvironmental factors (TPOM) framework.(23,24) The decision was taken to use the version of the NASSS framework produced by the aforementioned best-fit framework synthesis as it represented a determinant framework specifically refined with clinical AI data.(15) Its role was to guide the analysis of data into groupings of factors which have been shown to be influential of implementation outcomes in prior research of healthcare innovation with technology. The index publication concerning NASSS and publications of subsequent work illustrate the potential to grade each of the 7 top-level domains as simple, complicated or complex.(21) This approach was not taken in the present work as it did not aim to evaluate an existent intervention or implementation effort, rather to inform the design of one. As such this kind of categorical scoring was not thought to be beneficial and risked reductionism and oversight of useful detail. Through the course of the qualitative systematic review work, the research team had also developed familiarity confidence with its use. The domains, subdomains and subthemes of this adapted NASSS framework were used to place the data directly into one of 63 codes. If the researcher felt data were not readily placed within this codebook, further adaption to the framework was possible and there was no obligation to fill each subdomain. Data were not double coded, i.e. they were assigned to the single most relevant subtheme even when they could be related to multiple. This focused approach to coding aimed to mitigate against the scale and variation of the dataset.

An iterative and participatory approach was taken to analysis, over the course of several meetings with the study’s reference and advisory groups. The primary researcher would independently review coded data, to provide narrative summaries of factors likely to influence the implementation of AI-enabled macula services. The primary researcher would then present these factors at these various meetings alongside supporting data to facilitate discussion and revision of their interpretation.

Once the narrative had been conversationally agreed, summaries of what might influence implementation and why were written for each of the 63 codes, accompanied by illustrative quotes (Supplement 5). With the aim of informing subsequent investigations to inform how implementation ought to be conducted, insights into factors that appeared to be actionable or contentious were prioritised as key findings to be expressed in headed sections reflecting each of the 7 top-level NASSS domains.(21) Implementation factors where tension appeared to exist between stakeholder groups, or voices in the study and reference advisory groups, were also prioritised for these findings.

The second phase of analysis aimed to propose an actionable AI-enabled intervention for the macular service studied. Fifty distinct theories, models and frameworks previously used in qualitative research of clinical AI were reviewed to identify one well-suited to this analytical goal.(25) The Fit between Individuals, Technology and Task (FITT) Framework was identified as a relatively simplistic, action-oriented model, focused upon elements of an intervention which are modifiable.(22,26) Two leading alternatives were Davis’ Technology Acceptance Model (TAM) and Sittig and Singh’s Sociotechnical model.(27,28)

Both are well suited to facilitating thoughtful intervention design as they promote consideration of different facets of an intervention and highlight consequences of those design choices that are likely to influence implementation. However, TAM focuses more heavily on individual attributes such as the behavioural and psychological responses to interventions by adopters, which can only be indirectly influenced by intervention design choices. Meanwhile, the Sociotechnical Model highlights numerous and diverse aspects of intervention characteristics and their implementation context. This is helpful in more fully understanding the aspects of an intervention that may influence its implementation but risks distraction with non-modifiable aspects of an intervention or the target context.

Following re-familiarisation with the primary data and first analysis phase, each of the three domains of Individuals, Task and Technology were then reviewed. Influential factors identified in the initial analysis which appeared related to one of these domains were used to shape an aspect of the intervention. When determinants relevant to a single aspect of the intervention were poorly aligned, an initial judgement was made by the lead researcher to compromise or prioritise between determinants. This process was completed independently by the lead researcher until a draft intervention was complete. This draft was then discussed with the study reference group to gain additional perspectives on intervention design and to support further iterations of the intervention. The aspects of the intervention which were based on conflicting data were made a particular focus for these discussions. Following this analysis, the proposed intervention was then presented and validated through parallel roundtable discussions at a face-to-face public engagement event in Newcastle upon Tyne on 4^th^ October 2023. This event was attended by 34 members of the public, ophthalmology patients, eye charity professionals, academics and clinicians.

## Study reference group

The initial patient and carer members of the TEMS study reference group were engaged through a local meeting of the Macula Society, a large UK charity, within 20 miles of Newcastle-upon-Tyne. These members supported the initial study design prior to the award of funding and throughout the work. External peer-review of the study protocol during the funding process highlighted the value for wider involvement of members of the public who could offer perspectives of potential future service users. An advertisement for an additional four members of the public, without prior experience of ophthalmology services, was placed on a public research engagement internet platform in the UK. These four individuals were drawn from across the UK and engaged through email exchange and video conference meetings.
