# Supplementary material for: Intervention design for artificial intelligence-enabled macular service implementation: a primary qualitative study
Source: Implement Sci Commun. 2024 Nov 26;5:131. doi: 10.1186/s43058-024-00667-9 (PMC11600873; doi:10.1186/s43058-024-00667-9)
Supplement: Supplementary file 4 — Supplementary Material 4. S4. Example topic guide. [file 43058_2024_667_MOESM4_ESM.docx]

**Current pathway**

1. What parts of the current service do you like most?
2. What changes would you like to see in the current service?
3. In the current service you meet lots of team members for different parts of your care. Which bits of face-to-face or written contact do you find most valuable?
4. Have you had any ‘injection only’ or virtual clinic visits, where a consultation isn’t part of the visit? If so, how do you find them?

**Initial impressions of clinical AI**

1. As you know, I’m interested in the idea of technology in healthcare. What are your first thoughts and feelings when I mention artificial intelligence in healthcare?
2. When I’m talking about AI here, I mean a type of technology designed to take in some information and make a decision about it on its own. Specifically, I’m talking about a kind of AI we have, which can look at your eye photos on its own and see when your eye next needs an injection without help from a nurse or doctor. What use could you imagine for AI like that?
3. What kind of down-sides or difficulties do you think there might be in using that kind of AI in the clinic?

**Pathway placement**

1. Who do you think the best person to be responsible for the AI would be?
2. What kind of interactions would you like to have with doctors if artificial intelligence is brought in?
3. Where would you like to have your eye photos taken?
4. Where would you like to have your injections given?

**Relationships with the tool and others**

1. Some people might feel a bit uncomfortable about letting AI take some of the responsibility for their treatment planning. What kind of things might help you trust AI like this?
2. With this kind of AI, you might be able to see how and why it makes its treatment decisions for you. How would you feel about that?
3. How might it change the way friends and family support you in managing your eye disease?
4. Who would you want to be able to access the AI’s decision making?
5. How do you think bringing this AI into the service would affect your relationships with different members of the care team?

**Closing**

1. Thanks very much for so many helpful insights. Is there anything else we haven’t talked about that seems important about using AI in macular degeneration clinics?
2. We’re planning on talking to hospital doctors and nurses, opticians and managers but do you think there are other people’s perspectives we should be hearing?
