# Supplementary material for: Intervention design for artificial intelligence-enabled macular service implementation: a primary qualitative study
Source: Implement Sci Commun. 2024 Nov 26;5:131. doi: 10.1186/s43058-024-00667-9 (PMC11600873; doi:10.1186/s43058-024-00667-9)
Supplement: Supplementary file 5 — Supplementary Material 5. S5. Example NASSS narrative summary. [file 43058_2024_667_MOESM5_ESM.docx]

#### 1a.1 Type or format of care need

Patients, clinicians and commissioners perceived a very high personal and economic burden from nAMD treatment. This comes from both the frequency of injections and the serious consequence of irreversible sight loss if that frequent need is not met. Coupled with the high prevalence of the condition, nAMD treatment was a high strategic priority for commissioners and hospital managers. There is also a sense that treatment would be improved if the act of scheduling and administering the treatment did not have to displace more patient-centred social, psychological and broader clinical considerations. At present, most of clinicians’ attention is diverted toward the relatively simplistic decision-making around when treatment is required for patients with established diagnoses:

*“…the two decisions that you need to make are, do they need an injection, and when do they need the next one?... You just look at the picture and say it’s dry, and they’ve had 10 weeks, let’s try 12 weeks… It’s as simple as that. So, you don’t need a person.”* [HCP4]

Here an ophthalmology trainee shares their frustration over the amount their time spent on producing these decisions around treatment timing, later stating “my daughter can tell you that”. Similarly, patients, clinicians and mangers felt that the delivery of the injections themselves is an inefficient use of ophthalmologists time. Both patients and HCPs expressed a preference to prioritise this time for consultations around diagnosis, screening for ocular co-morbidities and changes to the management plan (e.g. cessation of injections).

#### 4a.2 Tools redefine staff roles

Most participants felt that AI-enabled nAMD treatment monitoring would change both the nature of work required and the staff groups best-suited to the work.

*“If you are deskilling them in a role that's no longer needed, well, that's not a problem. We no longer need the person who looks after leeches. That's not a big deal*.“ [HCP 11]

This GP hints at the extreme end of this spectrum with potential redundancy for eye specialists to be involved in nAMD treatment monitoring. Some participants felt an unfair professional threat for themselves or others, given the personal investments HCPs had made to achieve their competencies. Patients felt these shifting roles could assign greater priority to discussion and empathy from their clinicians. Most HCPs expected the potential for role expansion through AI-enabled care to increase the value they could contribute to patient care. Commissioners voiced things more pragmatically but seemed to welcome the opportunity to reduce staffing requirements for care provision. Most participants caveated their contributions, recognising that the exact implications of AI adoption would depend upon the detail of the use case.
