# Supplementary material for: Intervention design for artificial intelligence-enabled macular service implementation: a primary qualitative study
Source: Implement Sci Commun. 2024 Nov 26;5:131. doi: 10.1186/s43058-024-00667-9 (PMC11600873; doi:10.1186/s43058-024-00667-9)
Supplement: Supplementary file 6 — Supplementary Material 6. S6. Engagement event outline. [file 43058_2024_667_MOESM6_ESM.docx]

Exploring Technologies for Future Wet Macular Degeneration Care

| **09.45** | Arrival with tea and coffee |
| --- | --- |
| **10.00** | Welcome and outline for the day (Jeff Hogg) |
| **10.10** | A patient perspective on wet macular degeneration (Trevor Lunn) |
| **10.20** | Wet macular degeneration service provision; challenges and opportunities (James Talks) |
| **10.30** | Introduction to the research project; challenges and opportunities for current care (Jeff Hogg) |
| **10.45** | Discussions on tables (facilitators) |
| **11.30** | REFRESHMENTS |
| **11.50** | Publicly funded research for artificial intelligence in healthcare (Ian Newington) |
| **12.00** | Research findings; how artificial intelligence might support treatment decisions (Jeff Hogg) |
| **12.15** | Discussions on tables (facilitators) |
| **13.00** | LUNCH |
| **13.45** | Feedback from morning’s discussions (Jeff Hogg) |
| **14.00** | Future treatments to slow the progress of advanced dry macular degeneration (Ajay Kotagiri) |
| **14.15** | Home OCT imaging and the opportunities for macular degeneration monitoring (Ben Hunt) |
| **14.25** | The impact of macular degeneration on people in the UK now and in the future (Lucinda Hardy) |
| **14.40** | Next steps for research and practice (Jeff Hogg) |
| **14:55** | Thanks and close (Jeff Hogg) |
